# Impact of 30° Reserve Trendelenburg Position on Lung Function in Morbidly Obese Patients Undergoing Laparoscopic Sleeve Gastrectomy

**DOI:** 10.3389/fsurg.2022.792697

**Published:** 2022-02-24

**Authors:** Dengyu Gao, Lu Sun, Ning Wang, Yubo Shi, Jianli Song, Xiaoying Liu, Qiyao Yang, Zhenbo Su

**Affiliations:** ^1^Department of Anesthesiology, China-Japan Union Hospital of Jilin University, Changchun, China; ^2^Department of Anesthesiology, The First Affiliated Hospital of Xiamen University, Xiamen, China; ^3^Education Centre, China Medical Tribun, Beijing, China

**Keywords:** laparoscopic sleeve gastrectomy, 30°-RTP, lung function, obese, obesity surgery

## Abstract

**Background:**

This study aimed to evaluate the impact of patients' positioning before and after intubation with mechanical ventilation, and after extubation on the lung function and blood oxygenation of patients with morbid obesity, who had a laparoscopic sleeve gastrectomy.

**Methods:**

Patients with morbid obesity (BMI ≥ 30 kg/m^2^, ASA I – II grade) who underwent laparoscopic sleeve gastrectomy at our hospital from June 2018 to January 2019 were enrolled in this prospective study. Before intubation, after intubation with mechanical ventilation, and after extubation, arterial blood was collected for blood oxygenation and gas analysis after posturing the patients at supine position or 30° reverse Trendelenburg position (30°-RTP).

**Results:**

A total of 15 patients with morbid obesity were enrolled in this self-compared study. Pulmonary shunt (Qs/Qt) after extubation was significantly lower at 30°-RTP (18.82 ± 3.60%) compared to that at supine position (17.13 ± 3.10%, *p* < 0.01). Patients' static lung compliance (Cstat), during mechanical ventilation, was significantly improved at 30°-RTP (36.8 ± 6.7) compared to that of those in a supine position (33.8 ± 7.3, *p* < 0.05). The PaO_2_ and oxygen index (OI) before and after intubation with mechanical ventilation were significantly higher at 30°-RTP compared to that at supine position, and in contrast, the P_A−a_O_2_ before and after intubation with mechanical ventilation was significantly reduced at 30°-RTP compared to that at supine position.

**Conclusion:**

During and after laparoscopic sleeve gastrectomy, patients with morbid obesity had improved lung function, reduced pulmonary shunt, reduced P_A−a_O_2_ difference, and increased PaO_2_ and oxygen index at 30°-RTP compared to that supine position.

## Introduction

The prevalence of obesity is increasing worldwide. It has been reported by the National Health and Nutritional Examination Survey (NHANES) that the prevalence of obesity in the USA was 40.4% for women and 35% for men in 2016 (1). In the European Union (EU) countries, approximately 40–50% of men and 25–35% of women were overweight and 15–25% of men and women were obese in 2008 (2). As a result of the current obesity epidemic, anesthetists are not the only ones challenged with anesthetizing patients with obesity. Pulmonologists are also confronted with properly ventilating these patients, along with maintaining normal lung function when they emerge from general anesthesia. This is due to patients with obesity having altered respiratory mechanics and metabolic syndrome/s (3, 4). In this regard, compared to a normal healthy person, significant alteration of respiratory mechanics exists in obesity, which is further augmented with general anesthesia or with underlying lung diseases. These alterations include a reduced functional residual capacity (FRC), reduced lung and chest wall compliance, increased lung resistance, reduced oxygenation, and increased work of breathing (5, 6).

Laparoscopic sleeve gastrectomy benefits patients with obesity by providing a quicker post-surgery recovery and shorter hospital stays (7–10). It has been reported that a minimally invasive laparoscopic surgery in patients with morbid obesity and with endometrial cancer was associated with fewer complications and fewer days of hospital stay compared to open surgery (11–13). However, the pneumoperitoneum and steep Trendelenburg position can cause a decrease in respiratory compliance, increase of intrathoracic pressure, and the reduction of functional residual volume (14–16). Consequently, postoperative pulmonary complications, such as atelectasis and mismatching of the ventilation/perfusion ratio, may occur in the patients undergoing laparoscopic abdominal surgery (17–20). All these pulmonary complications are further complicated in patients with morbid obesity, hence, anesthetic care for these patients during and after laparoscopic surgery is more challenging, not only to the anesthesiologists but also to the surgeons and pulmonologists. Currently, the following strategies have been explored to improve the lung functions of the patients with morbid obesity during and after laparoscopic abdominal surgeries: (1) Increase tidal volume, (2) Positive end-expiratory pressure (PEEP), and (3) Vital capacity maneuver (VCM) (21, 22). However, to our knowledge, the studies on the impact of patient's body position change on pulmonary functions and respiratory mechanics in patients with obesity, who have laparoscopic sleeve gastrectomy has not been reported, although the impact of the body position on surgery procedure *per se* has been reported (20, 23). Therefore, the current study was designed to investigate if 30° reverse Trendelenburg position (30°-RTP) before and after intubation with mechanical ventilation as well as after extubation could improve the patient's oxygenation and lung function compared to supine position.

## Methods

### Patient Enrollment

Inclusion criteria are as follows: (1) Patients who had laparoscopic sleeve gastrectomy in our hospital from June 2018 to January 2019, (2) patients with American Society of Anesthesiologists (ASA) grade I or II classifications. (3) patients with a body mass index (BMI) ≥ 30 kg/m^2^, and (4) patients who have signed a written consent form.

Exclusion criteria are as follows: (1) Patients who have a history of severe cardiovascular diseases, such as heart failure, myocardial infarction, arrhythmia, or severe hypovolemia; (2) patients who had a history of cerebrovascular diseases, such as intracranial hypertension, cerebral infarction, cerebral hemorrhage, carotid artery stenosis, or cerebral ischemia; (3) Patients who had a history of chronic pulmonary diseases, such as chronic obstructive pulmonary disease, asthma, pulmonary bullae, or respiratory failure; (4) Patients with severe anemia, Hb < 90 g/L; and 5) Patients who had pneumonia within 30 days prior to the surgery.

### Anesthesia

Patients stopped eating at 12 h and drinking at 6 h prior to the surgery. After a peripheral venous access setup, a Dash 5000 monitor was connected to obtain the patients' physiologic data that were included in the electrocardiogram and blood oxygenation index. Blood pressure was measured through radial artery puncture and catheterization.

The patient was at supine position and given the following medicines by intravenous infusion: midazolam (0.12 mg/kg), propofol (1.5 mg/kg), cis-atracurium (0.3 mg/kg), and sufentanil (0.7 μg/kg). Mechanical ventilation was then set as a pressure-controlled mode with 25–35 cm H_2_O airway pressure and 30–45 mmHg end-tidal partial pressure of carbon dioxide (PCO_2_). Sevoflurane was continuously inhaled during the operation and minimum alveolar concentration (MAC) was kept at 1.2–2 range. Central vein puncture and catheterization were performed, and cis-atracurium and sufentanil were periodically applied during the surgery to keep the desired anesthesia. Sevoflurane inhalation was stopped once the surgery was completed.

### Blood Gas Analysis at Three Different Time Points

1). Pre-intubation: upon entering the operation room, the patient was put at supine position and given high flow oxygen (8 L/min) for 10 min followed by an arterial blood gas analysis. Then, the patient was postured at 30°-RTP and was given high flow oxygen (8 L/min) for 10 min followed by arterial blood gas analysis again.

2). On-ventilation: After completion of the surgery, the patient was transferred to ICU with the support of mechanical ventilation at volume-controlled mode: 8 ml/kg tidal volume, 5 cm H2O PEEP, and 50% fraction of inspired oxygen (FiO_2_). Once the patient had stable circulation, arterial blood gas analysis and central vein blood gas analysis were performed at supine position. The patient's position was then changed to 30°-RTP for 10 min followed by arterial and venous blood gas analysis.

3). Post-extubation: Patients emerged from general anesthesia in the ICU at supine position. After extubation, oxygen (50% FiO_2_) was delivered with a Venturi mask for 10 min followed by arterial and venous gas analysis. The patient's position was then changed at 30°-RTP for 10 min followed by arterial and venous blood gas analysis again.

Blood gas analysis included the following parameters, which were collected before the surgery: partial pressure of oxygen (P_a_O_2_), oxygen index (OI), and alveolar-arterial oxygen tension difference (P_A−a_O_2_) at two different positions. Pulmonary shunt fraction (Qs/Qt), OI, P_a_O_2_, static lung compliance (Cstat), and (P_A−a_O_2_), in two different positions, were collected after surgery when the patients were intubated with mechanical ventilation. The Qs/Qt, OI, P_a_O_2_, and P_A−a_O_2_ at two different positions after extubation were collected and analyzed.

### Statistical Analysis

A preliminary study revealed that at 30°-RTP, airway resistance was reduced, hypoxia duration was prolonged, and tidal volume with face mask ventilation increased over 40%. This study was a self-compared and prospective clinical study. By the criteria of two-tailed a = 0.05 significance level and 80% confidence range, at least 12 participants were required to have sufficient statistical power for this study. Accordingly, a total of 15 patients was enrolled with the prediction of 20% failure. After the normality test, Student's *t-*test was used for comparison and *p* < 0.05 was considered as significant.

## Results

A total of 15 patients were enrolled in this study with the following demographic features: average age of 38 (18–52) years old; gender ratio of 9/6 (M/F); average height of 170 (159–182) cm; average weight of 108 (92–130) kg; average body mass index (BMI) of 37.4 (32.7–54) kg/m^2^; and ASA (I/II): 10/5.

As shown in Table 1, there was no significant difference in Qs/Qt between the two positions when the patients were on mechanical ventilation. However, in 10 min after extubation, Qs/Qt was significantly lower at 30°-RTP (18.82 ± 3.60%) compared to that at supine position (17.13 ± 3.10%, *p* <0.01). In addition, patients' static lung compliance (Cstat) during mechanical ventilation was significantly improved at 30°-RTP (36.8 ± 6.7%) compared to that at supine position (33.8 ± 7.3%, *p* < 0.01).

**Table 1:** Comparison of Qs/Qt (%) on mechanical ventilation and post-extubation.

**Positions**	**On MV (*N* = 15)**	**Post-extubation (*N* = 15)**
Supine position	17.76 ± 3.40	18.82 ± 3.60
30°-RTP	17.01 ± 2.90	17.13 ± 3.10^**^

*Qs/Qt: pulmonary shut fraction. MV, mechanical ventilation; RTP, Reverse Trendelenburg position*.

** *P < 0.01 compared to supine position*.

The partial pressure of oxygen (PaO_2_) at pre-intubation and mechanical ventilation were significantly improved at 30°-RTP compared to that at supine position (*p* < 0.05 or 0.01, Table 2). In addition, in either position, the PaO_2_ was significantly reduced when the patients were on mechanical ventilation or after 10 min post-extubation compared to pre-intubation (*p* < 0.01, Table 2). Similarly, oxygen index (OI) at pre-intubation and on mechanical ventilation was significantly higher at 30°-RTP compared to that at supine position (*p* < 0.05 or 0.01, Table 3). At either position, OI was significantly reduced when the patients were on mechanical ventilation (30% reduction) or after 10 min post-extubation (50% reduction) compared to that of pre-intubation (*p* < 0.01, Table 3).

**Table 2:** PaO_2_ (mmHg) at pre-intubation, on mechanical ventilation, and post-extubation.

**Positions**	**Pre-intubation** **(*N* = 15)**	**On MV** **(*N* = 15)**	**Post-extubation** **(*N* = 15)**
Supine position	243.1 ± 26.6	168.5 ± 42.1^##^	122.7 ± 35.5^##^
30°-RTP	262.5 ± 31.2^*^	189.5 ± 47.3^**^^##^	126.8 ± 47.5^##^

*PaO_2_, partial pressure of oxygen; MV, mechanical ventilation; RTP, Reverse Trendelenburg position*.

* *P < 0.05 or*

** *P < 0.01 compared to supine position*;

## *P < 0.01 compared to pre-intubation*.

**Table 3:** OI at pre-intubation, on mechanical ventilation, and post-extubation.

**Positions**	**Pre-intubation** **(*N* = 15)**	**On MV** **(*N* = 15)**	**Post-extubation** **(*N* = 15)**
Supine position	486.1 ± 53.2	337.1 ± 84.1^##^	245.5 ± 70.9^##^
30°-RTP	525.1 ± 62.4^*^	378.9 ± 94.7^**^^##^	253.6 ± 94.9^##^

*OI, oxygen index; MV, mechanical ventilation; RTP, Reverse Trendelenburg position*.

* *P < 0.05 or*

** *P < 0.01 compared to supine position*;

## *P < 0.01 compared to pre-intubation*.

In contrast, P_A−a_O_2_ at pre-intubation and on mechanical ventilation was significantly reduced at 30°-RTP compared to that at supine position (*p* < 0.05, Table 4). At either position, P_A−a_O_2_ was significantly increased when the patients were on mechanical ventilation or after 10 min post-extubation compared to that of pre-intubation (*P* < 0.01, Table 4).

**Table 4:** P_A−a_O_2_ at pre-intubation, on mechanical ventilation, and post-extubation.

**Positions**	**Pre-intubation** **(*N* = 15)**	**On MV** **(*N* = 15)**	**Post-extubation** **(*N* = 15)**
Supine position	73.5 ± 27.5	137.5 ± 43.1^##^	180.8 ± 37.0^##^
30°-RTP	50.7 ± 32.0^*^	118.7 ± 47.6^*^^##^	178.9 ± 49.1^##^

*P_A−a_O_2_: alveolar-arterial oxygen tension difference*.

*MV, mechanical ventilation; RTP, Reverse Trendelenburg position*.

* *P < 0.05 compared to supine position*;

## *P < 0.01 compared to pre-surgery*.

## Discussion

In the current study, the impact of 30°-RTP on lung function in patients with obesity, who had sleeve gastrectomy through laparoscopy, was compared to that at supine position. It was found that post-extubation Qs/Qt was significantly lower at 30°-RTP compared to that at supine position, that the patients' static lung compliance (Cstat) during mechanical ventilation was significantly improved at 30°-RTP compared to that at supine position, that PaO_2_ and oxygen index before and after intubation for mechanical ventilation were significantly higher at 30°-RTP compared to that at supine position, and, in contrast, the P_A−a_O_2_ before and after intubation for mechanical ventilation was significantly reduced at 30°-RTP compared to that at supine position. These findings suggested that when patients with obesity had laparoscopic gastrectomy, the 30°-RTP could improve the static lung compliance, reduce pulmonary shunt, reduce P_A−a_O_2_ difference, and increase PaO_2_ and oxygenation compared to that at supine position.

When a normal-weighted patient undergoes laparoscopic surgery, there are no significant changes in respiratory homeostasis. However, in patients with morbid obesity, pulmonary function is significantly affected by CO_2_ pneumoperitoneum and by the movement of intra-abdominal contents against the diaphragm during the surgery and even after surgery. The major respiratory complications during and after laparoscopic surgery in patients with obesity include the following: (1) lung volume reduction, or atelectasis, with or without small airway closing; (2) decreased pulmonary or chest wall compliance; and (3) moderate to severe hypoxemia (24). These adverse respiratory effects are due to a Trendelenburg position during the surgery in addition to pneumoperitoneum and pressure on the diaphragm. Therefore, laparoscopic surgery for patients with morbid obesity is a special challenge for anesthesiologists, surgeons, and the entire perioperative care team. The following strategies have been applied to reduce the adverse effects on lung function in patients with obesity undergoing anesthesia and surgery under laparoscopy: (1) Application of PEEP and (2) Lung recruitment maneuver, that is, a small airway opening by applying intermittent hyperinflation of the lung (21). However, to our knowledge, while limited studies have been reported to explore the impact of the patient's body position on laparoscopic surgery procedure *per se* (20, 23), studies on the effect of changing the patient's position on lung function during and after laparoscopic sleeve gastrectomy has not been reported. In the current study, we found that the lung function of patients with obesity, during and after laparoscopic sleeve gastrectomy, was improved when the patients were at 30°-RTP compared to that at supine position. Consistently, studies have reported that a 25° head and trunk elevation resulted in a slower decrease in PaO_2_ following pre-oxygenation (25, 26).

Increased fat within the chest and abdominal wall causes a decrease in lung and chest wall compliance, increased airway resistance, and a reduced FRC (3, 21, 27). Closure of the airways leads to alveolar collapse, ventilation/perfusion mismatch, and hypoxia caused by pulmonary shunting (28, 29). In this self-controlled study, artery blood was collected and analyzed before and after changing from supine position to 30°-RTP in patients with morbid obesity. Compared to that at supine position, arterial PaO_2_ and oxygen index were increased at 30°-RTP. Despite this, arterial PaO_2_ and oxygen index were reduced when the patients were on mechanical ventilation under anesthesia, and further reduced after extubation. These findings suggested that optimization of lung function is likely to be achieved by a posture that improves respiratory mechanics, lung volumes, and arterial oxygen tension after intubation and on mechanical ventilation, as well as during the emergence from general anesthesia.

The main cause of ventilation-perfusion mismatch in obesity is underventilation of well-perfused lower lung regions (30). This change in respiratory mechanics increases the possibility of developing pulmonary complications in patients with obesity, especially when they have laparoscopic surgery. It has been reported that atelectasis and pneumonia were the two leading postoperative adverse events following a bariatric surgery under laparoscopy (31, 32). Moreover, morbidly patients with obesity have persistent atelectasis not only during general anesthesia and abdominal surgery but also postoperatively compared to their non-obese counterparts (33, 34). Weight loss through bariatric surgery could partially prevent the development of such pulmonary complications by increasing the expiratory reserve volume (35). In the current study, before extubation, patients' PaO_2_, OI, and P_A−a_O_2_ were significantly higher at 30°-RTP than in supine position. After extubation, however, there were no significant differences between the two positions in the aforementioned parameters. This could be due to patients falling asleep and airway collapse following extubation.

Pulmonary shunt (Qs/Qt) is 2–6% in a normal healthy person, but it significantly changes in obesity. It is further exacerbated to be as high as 20% with general anesthesia and mechanical ventilation. In the current study, Qs/Qt was 17% when the patients with obesity were intubated and on mechanical ventilation. After extubation in supine position though, it significantly increased (>18%), but not at 30°-RTP. These findings suggested that severe oxygen desaturations and pulmonary shunt can be avoided by optimizing the position of the patient with the head and trunk elevation.

There were limitations of this study. First of all, only 15 cases of patients who were morbidly obese were enrolled in this study. Hence, it is recommended that future studies have a larger population. Second, this was a self-comparative study. While an advantage of this study design was that we could archive reliable results with a limited number of cases, a prospective cohort study in the future would be helpful to further confirm the findings of this study. Third, the patients enrolled in this study were only given “sleeve gastrectomy,” although other surgical methods such as Roux-en-Y gastric bypass have also been used for the treatment of morbid obesity (12). Lastly, comparison of 30°-RTP in patients with morbid obesity who underwent laparoscopic sleeve gastrectomy vs. Roux-en-Y gastric bypass remains to be conducted in future studies.

In conclusion, the current study demonstrated that the position of 30°-RTP was readily achievable in patients with morbid obesity during laparoscopic sleeve gastrectomy with mechanical ventilation. It also demonstrated that patients' static lung compliance (Cstat) during mechanical ventilation significantly improved at 30°-RTP compared to that at supine position. In addition, the patient's pulmonary shunt (Qs/Qt) and P_A−a_O_2_ were significantly reduced, while PaO_2_ and oxygen indexes were significantly increased at 30°-RTP compared to that at supine position. These findings suggested that a short period of 30°-RTP during and after laparoscopic sleeve gastrectomy could significantly improve lung functions in patients with morbid obesity.

## Data Availability Statement

The original contributions presented in the study are included in the article/supplementary material, further inquiries can be directed to the corresponding author.

## Ethics Statement

The studies involving human participants were reviewed and approved by the Ethics Committee of China-Japan Union Hospital of Jilin University. The patients/participants provided their written informed consent to participate in this study.

## Author Contributions

DG, LS, and ZS contributed to the study conception and design. All authors collected the data, performed the data analysis, contributed to the interpretation of the data, the completion of figures and tables, contributed to the drafting of the article and final approval of the submitted version.

## Funding

This work was supported by the Education Department of Jilin Province (No. JJKH20170862KJ) and the Science and Technology Department of Jilin Province (No. 20190701068GH).

## Conflict of Interest

The authors declare that the research was conducted in the absence of any commercial or financial relationships that could be construed as a potential conflict of interest.

## Publisher's Note

All claims expressed in this article are solely those of the authors and do not necessarily represent those of their affiliated organizations, or those of the publisher, the editors and the reviewers. Any product that may be evaluated in this article, or claim that may be made by its manufacturer, is not guaranteed or endorsed by the publisher.
